# Michael hydratase alcohol dehydrogenase or just alcohol dehydrogenase?

**DOI:** 10.1186/s13568-014-0030-2

**Published:** 2014-03-15

**Authors:** Verena Resch, Jianfeng Jin, Bi-Shuang Chen, Ulf Hanefeld

**Affiliations:** 1 Biocatalysis, Department of Biotechnology, Delft University of Technology, Julianalaan 136, Delft, 2628 BL, The Netherlands

**Keywords:** Michael addition, α,β-Unsaturated carbonyl compounds, Hydratase, Alcohol dehydrogenase

## Abstract

The Michael hydratase – alcohol dehydrogenase (MhyADH) from *Alicycliphilus denitrificans* was previously identified as a bi-functional enzyme performing a hydration of α,β-unsaturated ketones and subsequent oxidation of the formed alcohols. The investigations of the bi-functionality were based on a spectrophotometric assay and an activity staining in a native gel of the dehydrogenase. New insights in the recently discovered organocatalytic Michael addition of water led to the conclusion that the previously performed experiments to identify MhyADH as a bi-functional enzyme and their results need to be reconsidered and the reliability of the methodology used needs to be critically evaluated.

## Introduction

The Michael addition of water to α,β-unsaturated ketones is a very interesting but rarely investigated reaction in organic synthesis and only a few procedures are described (Riedel and Krekeler [1972]; Mahajan et al. [2006]; Wang et al. [2006]; Boersma et al. [2010a]; Boersma et al. [2010b]). In contrast to the low number of chemical methods, nature is capable of performing this reaction and many examples have been described (Jin and Hanefeld [2011]). The beauty of this reaction is obvious: Using water as a nucleophile and solvent would allow a green route to hydroxy ketones. Hydratases as biocatalysts would allow an even more environmentally benign approach. Prominent and well studies examples are fumarase, malease, citraconase and enoyl-CoA hydratase. One drawback of the enzymes described is their narrow substrate scope caused by their involvement in (primary) metabolic pathways where specificity is highly important. For their optimal use in biocatalysis, this ability is not desired and therefore new hydratases with a broader substrate spectrum are required. An remarkable example is the use of whole cells from *Rhodococcus rhodochrous* to catalyse the addition of water to 3-methyl- or 3-ethyl-2-butenolide reported by Holland and Gu ([1998]). Even though no extensive study on the substrate acceptance is reported and the hydratase involved in this transformation is not further characterised, the results are still promising for future investigations on hydratases with a broader substrate scope.

**Figure 1:**
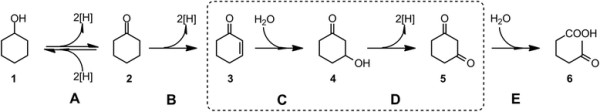
**Anaerobic degradation of cyclohexanol by*A. denitrificans*DSMZ 14773.** Compounds: **1** cyclohexanol, **2** cyclohexanone, **3** 2-cyclohexenone, **4** 3-hydroxycyclohexanone, **5** 1,3-cyclohexanedione, **6** 5-oxohexanoic acid. Enzymes: **A** cyclohexanol dehydrogenase; **B** cyclohexanone dehydrogenase; **C** 2-cyclohexenone hydratase; **D** 3-hydroxycyclohexanone dehydrogenase, **E** 1,3-cyclohexanedione hydrolase. The figure is based on the pathway proposed by Dangel et al. ([1989]).

One interesting source of potential hydratases are for example bacteria present in waste water treatment facilities. Waste water is often polluted with man-made chemicals and bacteria can adopt to this situation and acquire the ability to degrade and detoxify their environment. Already in 1988 and 1989 Georg Fuchs and co-workers reported investigations on a denitrifying bacterium from the *Pseudomonas* genus that is capable of degrading cyclohexanol (Dangel et al. [1988,1989]). In more recent studies they continued their investigations on denitrifying bacteria and results on the organism *Alicycliphilus denitrificans* (Oosterkamp et al. [2011,2013]; Weelink et al. [2008]) are reported (Mechichi et al. [2003]). The proposed degradation pathway for cyclic compounds starting from cyclohexanol and the enzymes involved are shown in Figure 1.

This degradation pathway also includes a potential hydratase (2-cyclohexenone hydratase) that is thought to perform the addition of water to 2-cyclohexenone followed by oxidation catalysed by a dehydrogenase to form 1,3-cyclohexanedione. To identify and characterise the enzymes involved in the pathway, a native PAGE gel staining was carried out (Dangel et al. [1989]).

Later experiments to find and investigate the hydratase described in this pathway were conducted by us, using *Alicycliphilus denitrificans* DSMZ 14773 as the bacterial source (Jin et al. [2010]; Jin et al. [2011]). The search for the hydratase was based on a spectrophotometric assay and the native PAGE gel staining method described by Dangel et al. in 1989. Results from these studies led to the conclusion that the hydratase is a bi-functional enzyme that also possesses the dehydrogenase activity if an electron acceptor is present. Therefore the identified enzyme was called Michael hydratase – alcohol dehydrogenase (MhyADH).

During the investigation on the Michael addition of water to 2-cyclohexenone a background reaction catalysed by proteinogenic amino acids was identified. This reaction was investigated further in recent studies (Resch et al. [2013]) that showed, that the reaction is in general base catalysed and that primary amines are good catalysts.

These new findings need to be incorporated into the search for MhyADH and their results indicate that the assumption that MhyADH is a bi-functional enzyme need to be reconsidered. Positive results gained from the spectrophotometric assay and the native PAGE staining might have led to false conclusions. Here we would like to report the new interpretation of the results and comment on the reliability of the assays used.

## Materials and methods

### Chemicals

Methylene blue, 2,6-dichlorophenol indophenol (DCPIP), and nitro blue tetrazolium chloride (NBT) were purchased from AcrosOrganics and 2-cyclohexenone and xanthine from Sigma Aldrich. KH_2_PO_4_ and MgSO_4_ • 7H_2_O were obtained from J. T. Baker. K_2_HPO_4_, NH_4_Cl, KNO_3_ and CaCl_2_ • 2H_2_O were purchased from Merck. Peptone, meat extract and agar were bought from BD.

### Microorganism and culture conditions

*A. denitrificans* DSMZ 14773 was purchased from the Deutsche Sammlung von Mikroorganismen und Zellkulturen (DSMZ, Germany). The reactivation medium contained: peptone (5.0 g/L), meat extract (3.0 g/L), agar (for solid medium, 15.0 g/L), distilled water (1000 mL). Preculture in reactivation medium was grown aerobically at 30°C with reciprocal shaking at 150 rpm.

Medium for anaerobic cultivation contained: Solution A: KH_2_PO_4_ (0.816 g/500 mL), K_2_HPO_4_ (5.920 g/500 mL), distilled water (500 mL). Solution B: NH_4_Cl (0.530 g/500 mL), MgSO_4_ • 7 H_2_O (0.200 g/500 mL), KNO_3_ (2.000 g/500 mL), CaCl_2_ • 2 H_2_O (0.025 g/500 mL), distilled water (500 mL). Solution A and B were autoclaved separately, mixed, and 10 mL of trace element solution SL-10 containing HCl (25%, 7.7 M, 10.00 mL), FeCl_2_ • 4 H_2_O (1.5 g/L), ZnCl_2_ (70 mg/L), MnCl_2_ • 4 H_2_O (100 mg/L), B(OH)_3_ (6.00 mg/L), CoCl_2_ • 6 H_2_O (190 mg/L), CuCl_2_ • 2 H_2_O (2.00 mg/L), NiCl_2_ • 6 H_2_O (24.00 mg/L), Na_2_MoO_4_ • 2 H_2_O (36.00 mg/L), distilled water (990 mL), followed by the addition of 5 mL of vitamin solution containing vitamin B_12_ (50 mg/L), pantothenic acid (50 mg/L), riboflavin (50 mg/L), pyridoxamine-HCl (10 mg/L), biotin (20 mg/L), folic acid (20 mg/L), nicotine amide (25 mg/L), nicotinic acid (25 mg/L), α-lipolic acid (50 mg/L), p-aminobenzoic acid (50 mg/L), and thiamine-HCl • 2 H_2_O (50 mg/L). Both trace element solution and vitamin solution were added using a sterile syringe filter. Medium was excessively degassed with argon, inoculated with preculture, and cyclohexanol (100 μL/L) was added as the carbon source. The culture was shaken reciprocally in air-tight 2 L bottles for 4 days at 150 rpm at 30°C. Cells were harvested by centrifugation and purified as previously described (Jin et al. [2011]). The cell-free extract of *A. denitrificans* was applied to a DEAE Sepharose column (28 mL) previously equilibrated with buffer A (20 mM Tris–HCl, pH 7.8). The elution was performed with buffer B (20 mM Tris–HCl, 1 M NaCl, pH 7.8) with a gradient from 0% to 50% at a flow rate of 1 mL/min.

TADH was produced according to previously described procedures (Höllrigl et al. [2008]; Hollmann et al. [2005]). *E. coli* BL21 (DE3) containing pASZ2 (pET 11a derivative containing the gene for TADH) was grown in Overnight Express TM Autoinduction System (Novagene) at 37°C for 24 h. Harvested cells were resuspended in buffer, broken using a French press and incubated at 80°C for 20 minutes. After ultracentrifugation the supernatant was used directly without further purification.

### Native PAGE and activity staining of MhyADH and TADH

Native polyacrylamide gel electrophoresis (PAGE) was performed using 4–15% gradient gels (Phast Gel system form GE Healthcare Life Sciences). Partially purified enzyme preparations solution (concentration of stocks: MhyADH = 1.5 mg/ml, TADH = 1.0 mg/mL) were applied on gel, and gels were run on an PhastSystem separation and control unit from Pharmacia using the predefined program for native gels at 12°C. For size determination a standard protein calibration kit (GE Healthcare Life Sciences) containing albumin (66 kDa), lactate dehydrogenase (140 kDa), catalase (232 kDa), ferritin (440 kDa), and thyroglobulin (669 kDa) was used. Activity staining was performed with 30 mL of 100 mM Tris–HCl (pH 7.8) containing 0.6 mM NAD^+^ (for TADH), 60 μM methylene blue (for 3-hydroxycyclohexanone dehydrogenase), and 0.3 mM nitro blue tetrazolium chloride. The staining was initiated with the addition of 1 mM 2-cyclohexenone or 3-hydroxycyclohexanone and was carried out at room temperature. As soon as blue bands were visible, the staining solution was replaced by water and destaining was performed for 1 h. Standard proteins and partly purified enzyme preparations were stained with SimplyBlue™ SafeStain (Novex) and destained with distilled water.

### Preparative activity tests

Reactions were carried out in 1.5 mL screw-capped glass vials to prevent evaporation of substrate/product. Shaking was performed in a heated table top shaker at 30°C. The reaction was buffered using 100 mM Tris–HCl buffer at pH 7.8 at a total volume of 1 mL containing 1.5 mg partially purified MhyADH. Substrate 2-cyclohexenone or xanthine (5 mg, 0.05 mmol, final concentration 50 mM) was added (when xanthine was used, DMSO was used to improve the solubility). For blank reactions the setup was the same without the addition of enzyme. Reactions were allowed to proceed at the given temperature for 3 h and 24 h. For work-up, the aqueous reaction mixtures were saturated with NaCl followed by extraction with ethyl acetate (2 × 0.5 mL). Combined organic layers were dried over Na_2_SO_4_ and analysed on GC.

The same test was applied for the fractions collected from the purification to track back the hydratase activity. For this activity test blanks were prepared as follows: 0.5 mL of the collected fraction was denaturated by heat at 99°C for 30 min. These samples were cooled down and threatened in the same way as the non denaturated samples. Samples were directly mixed with substrate (2-cyclohexenone, 5 mg, 0.04 mmol) and shaken at 30°C for 3 h. The reactions were stopped by the addition of NaCl to ensure a saturated solution followed by the extraction with EtOAc (2 times 0.5 mL). Combined organic phases were dried with Na_2_SO_4_ and analysed on GC. Dodecane was used as an internal standard.

### Synthesis of 3-hydroxycyclohexanone

Since substrate 2-cyclohexenone is commercially available, only 3-hydroxycyclohexanone needed to be synthesised following a previously described procedure (Karmee et al. [2011]). A solution of K_2_Cr_2_O_7_ (3.74 g, 12.0 mmol) in conc. H_2_SO_4_ (3 mL) and water (15 mL) was added dropwise to a stirred solution of cyclohexane-1,3-diol (4.34 g, 37.3 mmol) in Et_2_O (15 mL). After the addition, the reaction was allowed to proceed at room temperature for 3 h. Work-up was performed by extraction with Et_2_O (5 × 30 mL). Combined organic layers were dried over Na_2_SO_4_ and solvents were evaporated under reduced pressure. Purification was performed using silica gel column chromatography (eluent system: petroleum ether: ethyl acetate = 1:1). 3-Hydroxycyclohexanone was obtained as a yellowish oil (2.14 g, 18.75 mmol, 50% yield). NMR (CDCl_3_, 400 MHz): *δ* (ppm) = 1.64–1.80 (2H, m); 1.96–2.11 (2H, m); 2.29 (2H, t, *J* = 6.6 Hz); 2.39 (1H, dd, *J* = 7.5 Hz, 14.1 Hz), 2.46 (1H, s), 2.63 (1H, dd, *J* = 4.1 Hz, 14.1 Hz); 4.14–4.21 (1H, m). ^13^C NMR (CDCl_3_, 100 MHz): *δ* (ppm) = 20.5, 32.4, 40.7, 50.1, 69.3, 210.8. Obtained NMR results were in accordance with literature (Karmee et al. [2011]). GC–MS (EI, 70 eV): *m*/*z* = 114 (M^+^, 24), 73 (13), 71 (33), 69 (13), 68 (45), 60 (46), 58 (30), 57 (19), 55 (66), 54 (15), 44 (99), 43 (100), 42 (94), 41 (48), 40 (13).

### Analytical methods

The spectrometric activity assay for MhyADH and TADH was carried out at room temperature in a quartz cuvette. The reaction mixture (0.9 mL) contained 1 mM substrate (either 2-cyclohexenone or 3-hydroxycyclohexanone) and 60 μM dichlorophenol indophenol (DCPIP) as the electron acceptor. The reaction system was buffered using a 100 mM Tris–HCl buffer at pH 7.8. The reaction was initiated by addition of 0.1 mL of enzyme solution (concentration of stocks: MhyADH = 1.5 mg/ml, TADH = 1.0 mg/mL). The spectrum of the reaction mixture were recorded on a Hewlett-Packard 8452A diode array spectrophotometer at an interval of 60 s and absorbance change at 578 nm (DCPIP, ε = 16.8 cm^−1^ mmol^−1^) was followed.

Conversion of 2-cyclohexenone to 3-hydroxycyclohexanone was determined by GC (column: CP-Wax 52 CB [Varian] 50 m × 0.53 mm × 2.0 μm) using an internal standard (dodecane) and calibration lines for both substrate (2-cyclohexenone) and product (3-hydroxycyclohexanone) with the following specifications and temperature program: Start 80°C, hold for 5 min, with a rate of 50°C/min to 140°C, hold for 3 min, with a rate of 50°C/min to 200°C, hold for 3 min, with a rate of 50°C/min to 250°C, hold for 1 min; injector temperature: 250°C, detector temperature: 270°C, total nitrogen flow: 20 mL/min; Retention times: 2-cyclohexenone: 7.22 min, 3-hydroxycyclohexanone: 12.90 min, dodecane: 3.89 min. GC–MS analysis was carried out using He as carrier gas and a Varian FactorFour VF-1 ms column (25 m × 0.25 mm × 0.4 μm), with detection by electron impact (EI) ionisation at 70 eV and quadrupole mass selection.

^1^H- and ^13^C NMR spectra were recorded on a 400 MHz spectrometer (^1^H: 400 MHz, ^13^C: 100 MHz) and chemical shifts (*δ*) are given in ppm. Column chromatography was performed using silica gel 60 (particle size 0.063–0.2 mm). Ethyl acetate and petroleum ether used for column chromatography were distilled before use. For TLC silica gel Plates 60 F_254_ were used.

## Results

### Staining of 3-hydroxycyclohexanone dehydrogenase activity in native gel

The results from previous experiments indicated that MhyADH is a bi-functional enzyme that possesses next to its hydratase activity also a dehydrogenase activity. This conclusion was based on the fact that the native gel staining gave positive results when either 2-cyclohexenone or 3-hydroxycyclohexanone were used in the experiment. Having gained more insight into the non-enzymatic water addition reaction, a more detailed study is necessary now. Since the hydration reaction of 2-cyclohexenone is a base-catalysed reaction and can take place spontaneously or catalysed by amino acids (Resch et al. [2013]) the staining experiment was repeated. Using an alcohol dehydrogenase (TADH, the thermostable alcohol dehydrogenase from *Thermus* sp. ATN1), which is known to convert cyclic substrates like 3-hydroxycyclohexanone, as a control. At the same time this enzyme is not described to have a hydratase activity and is therefore suited to probe the reliability of the assay. TADH has a homotetrameric structure with a molecular weight of around 149 kDa (Höllrigl et al. [2008]) MhyADH was described as a heterotrimeric enzyme comprised of three subunits with an approximate molecular weight of 20, 30, and 90 kDa, respectively (Jin et al. [2011]).

**Figure 2:**
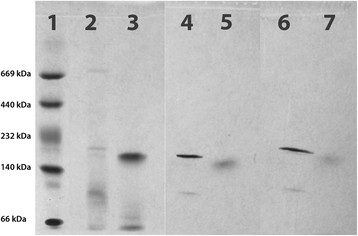
**Native gel activity staining.** Lane 1: molecular weight standard albumin (66 kDa), lactate dehydrogenase (140 kDa), catalase (232 kDa), ferritin (440 kDa), and thyroglobulin (669 kDa) stained with SimplyBlue™ SafeStain; lane 2: partially purified of MhyADH stained with SimplyBlue™ SafeStain; lane 3: partially purified TADH stained with SimplyBlue™ SafeStain; lane 4: partially purified MhyADH activity staining using 3-hydroxycyclohexanone as the substrate; lane 5: partially purified TADH activity staining using 3-hydroxycyclohexanone as the substrate; lane 6: partially purified MhyADH activity staining using 2-cyclohexenone as the substrate; lane 7: partially purified TADH activity staining using 2-cyclohexenone as the substrate.

The native activity staining was started using 2-cyclohexenone or 3-hydroxycyclohexanone as substrates to stain the MhyADH and the TADH band. In the case of 3-hydroxycyclohexanone as a substrate both bands are expected to be stained. Indeed, this is the case. Starting from 2-cyclohexenone only the MhyADH band should be stained due to its bi-functionality. If, however, the recently revealed chemical base-catalysed reaction takes place, both bands should be stained. Contrary to our initial assumption, this is the case and both TADH and MhyADH are stained and a false positive result is observed. In all cases – no matter whether 2-cyclohexenone or 3-hydroxycyclohexanone were used as substrates, both the bands for MhyADH and TADH were stained, indicating that the chemical water addition background reaction is fast enough to provide sufficient amounts of 3-hydroxycyclohexanone to serve as a substrate for the conversion to 1,3-cyclohexanedione and which resulted in false positive results for hydratase activity (Figure 2).

### Spectrophotometric assay

**Figure 3:**
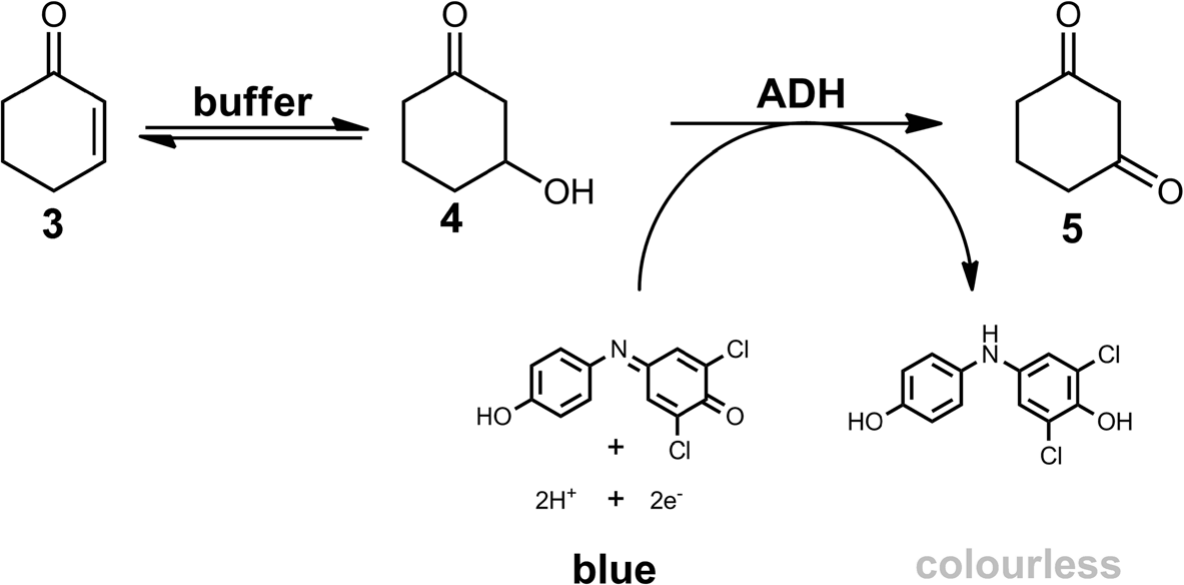
**Principle of the spectrophotometric assay.** The water addition to **3** is taking place spontaneously and the formed **4** is further converted by the ADH activity. The oxidised state of DCPIP is blue, the reduced colourless. DCPIP serves as an electron acceptor for the ADH, and its reduction can be detected as a decrease of absorbance. Results from the spectrophotometric assay using DCPIP are given in Table 1.

The results gained from the native PAGE staining were also reproduced in the colourimetric assay that was previously employed to test the substrate acceptance of the proposed hydratase (Dangel et al. [1989]; Jin et al. [2010]; Jin et al. [2011]). TADH was again used as a reference enzyme. The reaction was spectrometrically followed over time detecting the decrease of absorbance caused by a colour change from blue to colourless in the course of DCPIP reduction (See Figure 3).

As shown in Table 1 and in Additional file 1 a significant decrease in absorbance is measured when MhyADH is incubated with either 2-cyclohexenone or 3-hydroxycyclohexanone (entry 6 and 7). However, also when TADH is incubated with 2-cyclohexenone, a fast decrease in the absorbance is detectable (entry 8). This experiment confirms the results of the gel staining, indicating that 3-hydroxycyclohexanone is formed spontaneously and is then further converted to 1,3-cyclohexanedione by the dehydrogenase activity. To ensure that there is no influence on the absorbance caused by the substrates or the enzyme preparations, control reactions of all possible combinations with DCPIP were performed and no decrease in absorbance was observed (entries 1–5).

**Table 1:** Results of the spectrophotometric assay

**Entry**	**Experiment**	**Δ Abs/min**^**[a]**^
1	DCPIP + buffer	0.0003
2	DCPIP + **3**	-0.0008
3	DCPIP + **4**	-0.0012
4	DCPIP + TADH	-0.0001
5	DCPIP + MhyADH	-0.0007
6	DCPIP + **3** + MhyADH	-0.0087
7	DCPIP + **4** + MhyADH	-0.0080
8	DCPIP + **3** + TADH	-0.0199

[a] Decrease of absorbance is given as Δ absorbance unit per min. **3**: 2-cyclohexenone, **4**: 3-hydroxycyclohexanone. The graph to the data shown in the table can be found in the Additional file 1.

### Activity tests for hydratase activity

In addition to the native gel activity staining we also conducted preparative tests using partly purified enzyme solution and no addition of electron acceptors to stop the reaction at the Michael addition product. If hydratase activity is present, this should lead to the formation of 3-hydroxycyclohexanone and no oxidation to 1,3-cyclohexanedione should take place. The detailed characterisation of the water addition reaction catalysed by amino acids also showed that the reaction is subject to an equilibrium that lies on the 2-cyclohexenone side rather than on the 3-hydroxycyclohexanone side. This leads to a maximal possible conversion of around 25%. (Stiles and Longroy [1961]; Resch et al. [2013]). Taking this into account, utilising an authentic product references, and the proper analytical system to follow the conversion, tests with partly purified MhyADH solution were performed. These experiments revealed that no water addition faster than the background caused by basic condition of the buffer took place, indicating that the hydratase activity is either not stable under these conditions and hence deactivated, or not present.

## Discussion

The degradation pathway of cyclohexanol in *A. denitrificans* describes an array of enzymes capable of dealing with the detoxification of this substance. This pathway includes a hydratase and an alcohol dehydrogenase catalysing the addition of water and the subsequent oxidation of the formed alcohol by an alcohol dehydrogenase (Dangel et al. [1988,1989]). Attempts to identify and make use of the hydratase described were done previously and the enzyme was identified with a bi-functional nature having both a hydratase and an alcohol dehydrogenase activity (Jin et al. [2010,2011]). The addition of water to α,β-unsaturated ketones is known as the Michael addition (Tokoroyama [2010]), where water serves as the nucleophile and therefore as the Michael donor. Ongoing research on the Michael addition of water using 2-cyclohexenone showed that this reaction is in general base catalysed and that also simple amino acids serve as catalysts using 2-cyclohexenone and structurally related compounds as substrates (Resch et al. [2013]). Based on this new insights, the results and the methodology used in previous studies to identify the hydratase from *A. denitrificans* as a bi-functional enzyme need to be reinterpreted.

With a more detailed understanding of the Michael addition of water to 2-cyclohexenone we also obtained more insight into the reliability of the activity assay used to find the hydratase. Knowing that the water addition can take place in a spontaneous manner, the significance of the spectrophotometric assay needs to be doubted. Several conclusions from the control experiments using TADH as a comparative enzyme – where no hydratase activity is described – can be drawn.

(a) MhyADH is not a bi-functional enzyme but a structurally demanding ADH; (b) MhyADH is a bi-functional enzyme but the hydratase activity is not stable when isolated; (c) Using the coupled assay with 2-cyclohexenone as a substrate does not give reliable hints for the presence of a hydratase; (d) Using native gel staining (based on the same principle) does not give reliable hints for the presence of a hydratase.

All these conclusions can now be applied to the proposed degradation pathway of cyclohexanol (Dangel et al. [1989]) as well. It is not clear if a hydratase needs to be present for the water addition step. The water addition might also occur spontaneously and even though the dehydrogenase that catalyses the formation of 1,3-cyclohexanedione might be enantioselective, due to the equilibrium and the resulting dynamic kinetic resolution *via* the prochiral 2-cyclohexenone the preferred enantiomer of 3-hydroxycyclohexanone is always present.

The biochemical characterisation of MhyADH performed previously, revealed that the enzyme is a heterotrimere comprised of three subunits of different size and each one harbouring a different co-factor. The large subunit contains a molybdopterin, the medium subunit a FAD and the small subunit a [2Fe-2S] cluster (Jin and Hanefeld [2011]). This particular enzyme architecture is also found in the molybdenum-containing hydroxylase family. Other members of this family are for example xanthine dehydrogenase (Dietzel et al. [2009]; Hille [1996]), quinoline 2-oxidoreductase (Bonin et al. [2004]; Hille [1996]), and nicotinic acid dehydrogenase (Schräder et al. [2002]; Hille [1996]). Enzymes from the xanthine oxidase family are capable of introducing a hydroxyl group to the substrate where water is used as a source of the oxygen atom (Howes et al. [1996]; Leimkühler et al. [2004]; Okamoto et al. [2004]; Bonam and Ludden [1987]). All these enzymes share a common structural feature; they are composed of three subunits of different size whereby each subunit holds a different cofactor. In order to learn more about the function of MhyADH and due to its close relation to xanthine dehydrogenase, MhyADH was also tested for its ability to convert xanthine. In these tests no activity towards this substrate was found. Looking on the structural complexity of MhyADH, the question on the function of the different subunits arises. Xanthin dehydrogenase, quinoline 2-oxidoreductase or nicotinic acid dehydrogenase all belong to the family of molybdenum-containing oxidoreductases and the reaction these enzymes are catalysing are – compared to their complex quaternary structure – all quite simple. The ADH involved in the degradation of cyclohexanol by *A. denitrificans* can therefore be assigned to the group of molybdenum-containing oxidoreductases being able to catalyse the oxidation of cyclic alcohols and therefore displaying a completely different substrate pattern than for example xanthine dehydrogenase.

The additional experiments performed show the importance of detailed knowledge of a reaction system. The Michael addition of water is a rarely investigated reaction and detailed characterization is hardly found in literature. To ensure its presents, additional investigations on the possible hydratase in *A. denitrificans* are necessary including variation of the growth conditions and a screening methodology different from the coupled spectrophotometric assay needs to be employed to minimize the risk of producing false positive results.

## Competing interests

The authors declare that they have no competing interests.

## Additional file

## Supplementary Material

Additional file 1: **A: Principle of the colorimetric assay.** The water addition is taking place spontaneously and the formed 2-cyclohexenone is further converted by the ADH activity. The oxidised state of DCPIP is blue, the reduced colourless. DCPIP serves as a redox donor for the ADH. In parallel DCPIP is reduced which can be detected as a decrease of absorption. **B:** Results from the colorimetric assay using DCPIP. All control reactions are represented as empty marks. Reactions containing either TADH or MhyADH are represented as filled marks. 1: 2-cyclohexenone, 2: 3-hydroxycyclohexanone. Click here for file
