# Supplementary material for: Michael hydratase alcohol dehydrogenase or just alcohol dehydrogenase?
Source: AMB Express. 2014 Mar 15;4:30. doi: 10.1186/s13568-014-0030-2 (PMC4052635; doi:10.1186/s13568-014-0030-2)
Supplement: Additional file 1: — A: Principle of the colorimetric assay. The water addition is taking place spontaneously and the formed 2-cyclohexenone is further converted by the ADH activity. The oxidised state of DCPIP is blue, the reduced colourless. DCPIP serves as a redox donor for the ADH. In parallel DCPIP is reduced which can be detected as a decrease of absorption. B: Results from the colorimetric assay using DCPIP. All control reactions are represented as empty marks. Reactions containing either TADH or MhyADH are represented as filled marks. 1: 2-cyclohexenone, 2: 3-hydroxycyclohexanone. [file s13568-014-0030-2-S1.docx]

Additional file 1

AMB Express

Michael Hydratase Alcohol Dehydrogenase or Just

Alcohol Dehydrogenase?

Verena Resch, Jianfeng Jin, Bi-Shuang Chen, Ulf Hanefeld

Biocatalysis,

Department of Biotechnology,

Delft University of Technology,

Julianalaan 136, 2628 BL, Delft,

The Netherlands

e-mail: v.a.resch@tudelft.nl

e-mail: u.hanefeld@tudelft.nl


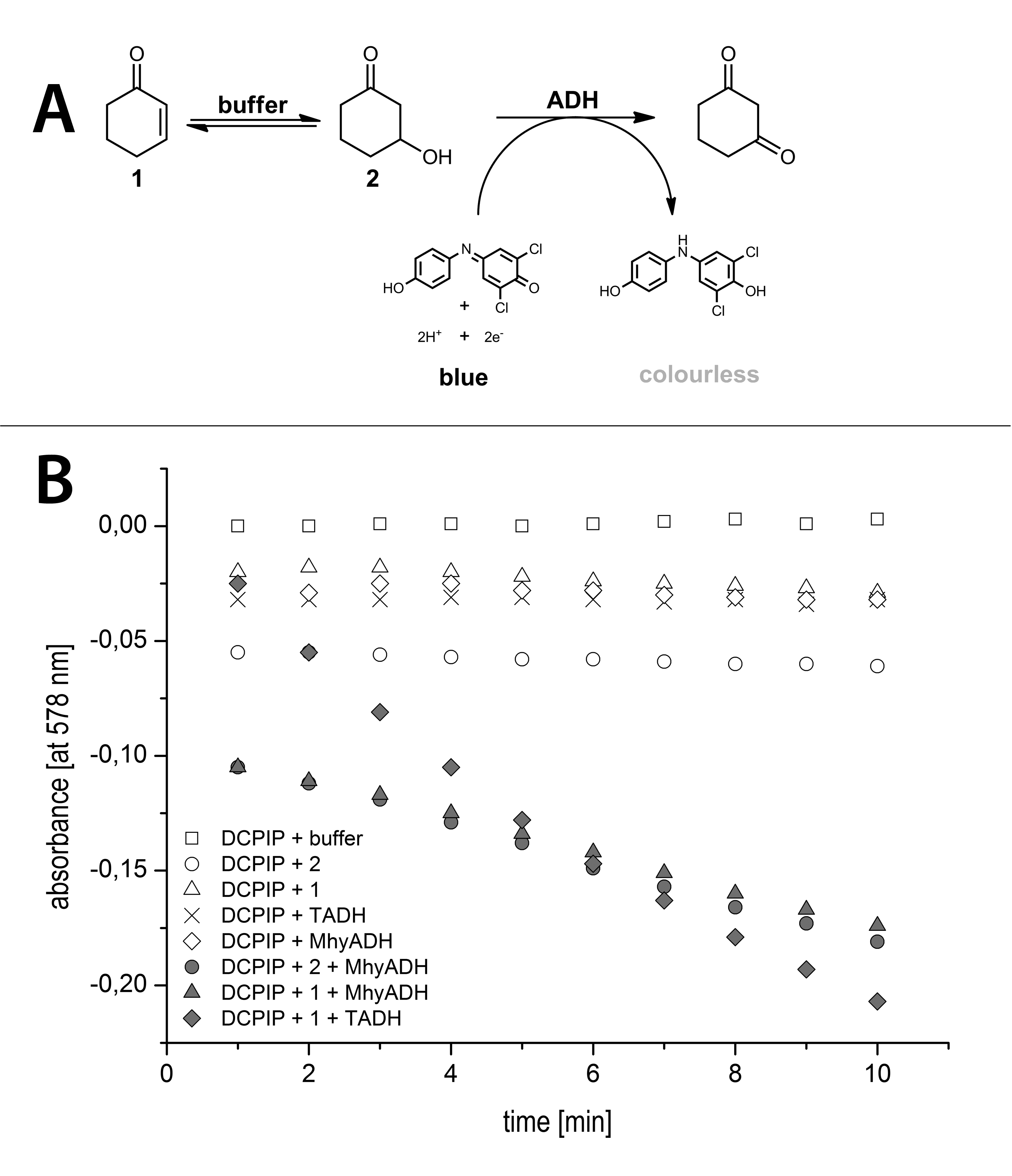


Fig. 1 A: Principle of the spectrophotometric assay. The water addition is taking place spontaneously and the formed 2-cyclohexenone is further converted by the ADH activity. The oxidised state of DCPIP is blue, the reduced colourless. DCPIP serves as a redox donor for the ADH. In parallel DCPIP is reduced which can be detected as a decrease of absorption. B: Results from the colorimetric assay using DCPIP. All control reactions are represented as empty marks. Reactions containing either TADH or MhyADH are represented as filled marks. 1: 2-cyclohexenone, 2: 3-hydroxycyclohexanone.
